# Optimizing the efficacy of exposure in PTSD treatment

**DOI:** 10.3402/ejpt.v6.27628

**Published:** 2015-04-08

**Authors:** Gert-Jan Hendriks, Rianne De Kleine, Agnes Van Minnen

**Affiliations:** 1 NijCare, Behavioural Science Institute, Radboud University Nijmegen, Nijmegen, The Netherlands; 2 Center for Anxiety Disorders Overwaal, Institution for Integrated Mental Health Care Pro Persona, Nijmegen, The Netherlands; 3 Department of Psychiatry, Radboud University Medical Centre, Nijmegen, The Netherlands

Both prolonged exposure (PE) and eye movement desensitization reprocessing (EMDR) are recommended in international guidelines as first choice treatments in PTSD patients. Its efficacy is showed in many randomized controlled trials and several meta-analytic reviews (Powers, Halpern, Ferenschak, Gillihan, & Foa, 2010). The proposed working mechanism in PE is fear extinction by effective emotional processing of traumatic memories. Notwithstanding the efficacy of PE, there is room for improvement. Many patients remain symptomatic after treatment (Schnurr et al., 2007), and dropout-rates are substantial and vary between 20 and 35% (Schnurr et al., 2014). Previous studies showed that adding psychological interventions to PE did not succeed in increasing effect-sizes compared to stand-alone PE (Kehle-Forbes et al., 2012). An alternative way to enhance PE efficacy is the combination with pharmacological treatment such as antidepressants, or oxytocin (see this issue: Olff et al., 2015). However, empirical data regarding the efficacy of combining PE with antidepressants are scarce. Additionally, the reported efficacy of adding antidepressants to PE varied between nil and modest compared to PE as stand-alone treatment (De Kleine, Rothbaum, & Van Minnen, 2013; Marshall et al., 2007; Rothbaum et al., 2006; Simon et al., 2008). On the other hand, compared to PE and EMDR the efficacy of pharmacological treatment as stand-alone in PTSD patients is likewise modest (Ipser, Seedat, & Stein, 2006; Watts et al., 2013). Approximately a decade ago an interesting new direction emerged. Fundamental research showed pharmacological enhancement of the underlying mechanisms in exposure therapy: extinction learning and reconsolidation (Debiec & Ledoux, 2004). Although the interrelation between extinction learning and reconsolidation is not fully understood, it is clear that these mechanisms are underlying the efficacy of exposure therapy (Kindt & Soeter, 2013). The findings in fundamental research were translated to clinical studies in anxiety disorders. As fear extinction is linked to the *N*-methyl-d-aspartate (NMDA) glutamatergic receptor activity in the basolateral amygdala, these studies focused on pharmacological enhancement of exposure therapy by NMDA receptor agonists, for instance d-cycloserine (DCS), patients with specific phobia, social anxiety disorder, panic disorder, and obsessive compulsive disorder (Bontempo, Panza, & Bloch, 2012; Norberg, Krystal, & Tolin, 2008). Studies in posttraumatic stress disorder were lacking. Therefore, we conducted a placebo-controlled randomized trial of DCS-enhancement PE treatment in a heterogeneous PTSD population (*n*=67). Although initially DCS-enhancement showed no difference compared to the placebo-group, we found that DCS enhanced PE treatment in a subgroup of patients with more severe PTSD at baseline and an initial non-response on PE (De Kleine, Hendriks, Kusters, Broekman, & Van Minnen, 2012). Furthermore, our results support the influence of personality traits on outcome. High consciousness and low extraversion predicted a better response in the DCS-enhancement treated patients compared to the placebo-group (De Kleine, Hendriks, Smits, Broekman, & Van Minnen, 2014). However, the results of DCS-enhancement in additional PTSD studies are controversial with conflicting results in outcome (Difede et al., 2014; Litz et al., 2012; Rothbaum et al., 2014). The proposed DCS-enhancement in exposure therapy as a one-size-fits-all enhancement strategy is unequivocally too optimistic.

## References

[CIT0001] Bontempo A, Panza K. E, Bloch M. H (2012). D-cycloserine augmentation of behavioral therapy for the treatment of anxiety disorders: A meta-analysis. The Journal of Clinical Psychiatry.

[CIT0002] Debiec J, Ledoux J. E (2004). Disruption of reconsolidation but not consolidation of auditory fear conditioning by noradrenergic blockade in the amygdala. Neuroscience.

[CIT0003] De Kleine R. A, Hendriks G. J, Kusters W. J, Broekman T. G, Van Minnen A (2012). A randomized placebo-controlled trial of D-cycloserine to enhance exposure therapy for posttraumatic stress disorder. Biological Psychiatry.

[CIT0004] De Kleine R. A, Hendriks G. J, Smits J. A, Broekman T. G, Van Minnen A (2014). Prescriptive variables for d-cycloserine augmentation of exposure therapy for posttraumatic stress disorder. Journal of Psychiatric Research.

[CIT0005] De Kleine R. A, Rothbaum B. O, Van Minnen A (2013). Pharmacological enhancement of exposure-based treatment in PTSD: A qualitative review. European Journal of Psychotraumatology.

[CIT0006] Difede J, Cukor J, Wyka K, Olden M, Hoffman H, Lee F. S (2014). D-cycloserine augmentation of exposure therapy for post-traumatic stress disorder: A pilot randomized clinical trial. Neuropsychopharmacology.

[CIT0007] Ipser J, Seedat S, Stein D. J (2006). Pharmacotherapy for post-traumatic stress disorder—A systematic review and meta-analysis. South African Medical Journal.

[CIT0008] Kehle-Forbes S. M, Polusny M. A, MacDonald R, Murdoch M, Meis L, Wilt T. J (2012). A systematic review of the efficacy of adding non exposure components to exposure therapy for posttraumatic stress disorder. Psychological Trauma: Theory, Research, Practice and Policy.

[CIT0009] Kindt M, Soeter M (2013). Reconsolidation in a human fear conditioning study: A test of extinction as updating mechanism. Biological Psychology.

[CIT0010] Litz B. T, Salters-Pedneault K, Steenkamp M. M, Hermos J. A, Bryant R. A, Otto M. W (2012). A randomized placebo-controlled trial of D-cycloserine and exposure therapy for posttraumatic stress disorder. Journal of Psychiatric Research.

[CIT0011] Marshall R. D, Lewis-Fernandez R, Blanco C, Simpson H. B, Lin S. H, Vermes D (2007). A controlled trial of paroxetine for chronic PTSD, dissociation, and interpersonal problems in mostly minority adults. Depression and Anxiety.

[CIT0012] Norberg M. M, Krystal J. H, Tolin D. F (2008). A meta-analysis of D-cycloserine and the facilitation of fear extinction and exposure therapy. Biological Psychiatry.

[CIT0013] Olff M, Van Zuiden M, Koch S, Nawijn L, Frijling J, Veltman D (2015). Intranasal oxytocin: Miracle cure after trauma?. European Journal of Psychotraumatology.

[CIT0014] Powers M. B, Halpern J. M, Ferenschak M. P, Gillihan S. J, Foa E. B (2010). A meta-analytic review of prolonged exposure for posttraumatic stress disorder. Clinical Psychology Review.

[CIT0015] Rothbaum B. O, Cahill S. P, Foa E. B, Davidson J. R, Compton J, Connor K. M (2006). Augmentation of sertraline with prolonged exposure in the treatment of posttraumatic stress disorder. Journal of Traumatic Stress.

[CIT0016] Rothbaum B. O, Price M, Jovanovic T, Norrholm S. D, Gerardi M, Dunlop B (2014). A randomized, double-blind evaluation of D-cycloserine or alprazolam combined with virtual reality exposure therapy for posttraumatic stress disorder in Iraq and Afghanistan war veterans. The American Journal of Psychiatry.

[CIT0017] Schnurr P. P, Chard K. M, Ruzek J. I, Chow B. K, Shih M. C, Resick P. A (2014). Design of VA Cooperative Study #591: CERV-PTSD, comparative effectiveness research in veterans with PTSD. Contemporary Clinical Trials.

[CIT0018] Schnurr P. P, Friedman M. J, Engel C. C, Foa E. B, Shea M. T, Chow B. K (2007). Cognitive behavioral therapy for posttraumatic stress disorder in women: A randomized controlled trial. JAMA.

[CIT0019] Simon N. M, Connor K. M, Lang A. J, Rauch S, Krulewicz S, LeBeau R. T (2008). Paroxetine CR augmentation for posttraumatic stress disorder refractory to prolonged exposure therapy. The Journal of Clinical Psychiatry.

[CIT0020] Watts B. V, Schnurr P. P, Mayo L, Young-Xu Y, Weeks W. B, Friedman M. J (2013). Meta-analysis of the efficacy of treatments for posttraumatic stress disorder. The Journal of Clinical Psychiatry.

